# Impact of co-administration of proton pump inhibitors on efficacy and safety of oral semaglutide in patients with type 2 diabetes: a single-center retrospective study

**DOI:** 10.1186/s40780-026-00617-8

**Published:** 2026-08-05

**Authors:** Tasuku Yokoyama, Tomonobu Uchino, Yasuharu Yazawa, Shohei Ueno, Daiki Tsuji

**Affiliations:** 1 Laboratory of Clinical Pharmaceutics, School of Pharmaceutical Sciences, University of Shizuoka, 52-1 Yada, Suruga-ku, Shizuoka City, Shizuoka 422-8526 Japan; 2 Laboratory of Clinical Pharmacokinetics, Research Support Center, Shizuoka General Hospital, 4-27-1 Kita-ando, Aoi-ku, Shizuoka City, Shizuoka 420-8527 Japan; 3 Laboratory of Clinical Pharmacology and Genetics, School of Pharmaceutical Sciences, University of Shizuokaï, 52-1 Yada, Suruga-ku, Shizuoka City, Shizuoka 422-8526 Japan; 4 Laboratory of Clinical Pharmacogenomics, Research Support Center, Shizuoka General Hospital, 4-27-1 Kita-ando, Aoi-ku, Shizuoka City, Shizuoka 420-8527 Japan

**Keywords:** Oral semaglutide, Proton pump inhibitor, Glycemic control, Body weight, Gastrointestinal adverse event

## Abstract

**Background:**

Oral semaglutide, a glucagon-like peptide-1 receptor agonist, improves glycemic control and reduces body weight in patients with type 2 diabetes. Although proton pump inhibitors (PPIs) have been reported to slightly increase the absorption of oral semaglutide, the impact of 24-week co-administration of PPIs on clinical efficacy and safety remains unclear.

**Methods:**

This retrospective study included patients with type 2 diabetes who initiated treatment with oral semaglutide between March 2021 and August 2024. The primary outcome was the change in the HbA1c at week 24. The secondary outcomes included the change in body weight and discontinuation of oral semaglutide due to gastrointestinal adverse events at week 24. Patients were divided into two groups according to PPI co-administration. Propensity score matching was performed to balance the baseline characteristics between the groups. Changes in HbA1c and body weight were analyzed using a linear mixed-effects model, and discontinuation rates were compared using Fisher’s exact test.

**Results:**

Among the 114 eligible patients, 30 received concomitant PPIs. After propensity score matching, 27 patients were included in each group. At week 24, the estimated mean change in HbA1c and body weight (95% confidence interval) was − 1.0% (-1.3 to -0.6) and − 1.4 kg (-2.6 to -0.3) in the PPI group and − 0.6% (-0.8 to -0.3) and − 1.8 kg (-2.5 to -1.0) in the non-PPI group. There were no statistically significant group-by-time interactions for HbA1c and body weight (*P* for interaction = 0.097 and 0.431, respectively). Discontinuation due to gastrointestinal adverse events did not differ significantly between the groups (25.9% vs. 18.5%).

**Conclusions:**

24-week co-administration of PPIs may not significantly affect the glycemic efficacy, weight reduction, or gastrointestinal tolerability of oral semaglutide. These findings suggest that the co-administration of PPIs is unlikely to meaningfully impact the clinical efficacy or tolerability of oral semaglutide in routine practice.

## Introduction

Glucagon-like peptide-1 receptor agonists (GLP-1 RAs) are recommended for the pharmacological management of type 2 diabetes because of their glycemic efficacy, cardiovascular benefits, and body weight reduction [1, 2]. Oral semaglutide is the first and currently the only oral formulation of GLP-1 RAs. To enhance its bioavailability, oral semaglutide contains the absorption enhancer sodium N-(8-[2-hydroxybenzoyl] amino) caprylate (SNAC) [3, 4]. SNAC has multiple complementary mechanisms, including local buffering of gastric acidity, protection from enzymatic degradation, maintenance of semaglutide in its monomeric form, and enhancement of transcellular absorption by increasing membrane fluidity [3, 4]. Therefore, the absorption of oral semaglutide depends on multiple gastric processes, and concomitant use of PPIs may influence its pharmacokinetics and potentially its clinical effects.

Proton pump inhibitors (PPIs) are widely prescribed for *H. pylori* eradication, acid-related disorders, and the prevention of gastrointestinal bleeding in patients receiving antiplatelet therapy [5, 6]. Long-term use of PPIs is common, particularly in patients with multiple comorbidities, and may increase the risk of clinically significant drug-drug interactions [7]. PPIs increase gastric pH and may influence drug solubility, drug release from pH-dependent formulations, and the bioavailability of prodrugs [8]. Accordingly, the co-administration of PPIs may influence the absorption of semaglutide. Baekdal et al. reported that the co-administration of a PPI increased semaglutide exposure by approximately 15% in healthy volunteers, although the difference was not statistically significant [9]. A recent exploratory study reported that co-administration of PPIs was associated with a greater reduction in HbA1c at week 52 in patients receiving oral semaglutide, whereas no significant difference was observed at week 26 [10]. Because the first 24–26 weeks represent a clinically important period for evaluating the early efficacy of oral semaglutide in pivotal clinical trials [3], these findings warrant further evaluation using more rigorous analytical methods.

We hypothesized that the co-administration of PPIs may alter the clinical effectiveness and safety of oral semaglutide. This study aimed to investigate the impact of 24-week co-administration of PPIs on glycemic control, body weight, and gastrointestinal adverse events in patients with type 2 diabetes treated with oral semaglutide.

## Materials and methods

### Study design

This retrospective study was conducted among outpatients at the Department of Diabetes and Endocrinology, Shizuoka General Hospital from March 2021 to August 2024. The inclusion criteria were as follows: (1) a diagnosis of type 2 diabetes and (2) baseline HbA1c between 7.0 and 9.5%, based on a previous randomized clinical trial [11]. Patients were initiated on 3 mg of oral semaglutide (Rybelsus^®^ tablets, Novo Nordisk Pharma Ltd., Tokyo, Japan), and the dose was increased to 7 mg after at least 4 weeks. The dose was further increased to 14 mg at the physician’s discretion if the treatment was effective and well tolerated. The exclusion criteria were as follows: (1) estimated glomerular filtration rate (eGFR) < 60 mL/min/1.73 m^2^, to minimize potential confounding in the assessment of gastrointestinal adverse events; (2) hospitalization due to acute infection or surgery during the observation period; (3) initiation of insulin or other oral antidiabetic medications within 30 days prior to initiation of oral semaglutide; and (4) poor adherence to oral semaglutide documented in the medical records. PPI use was defined as regular daily administration at the initiation of oral semaglutide, which continued for 24 weeks based on prescription records. We collected patient information, including age, sex, diabetes duration, body weight, body mass index (BMI), HbA1c, serum creatinine, eGFR, aspartate aminotransferase (AST), alanine aminotransferase (ALT), prior antidiabetic treatment, whether oral semaglutide was initiated as add-on or as a switch from other antidiabetic medications, and concomitant medications other than antidiabetic agents at baseline. Patients were followed up for 24 weeks after treatment initiation. The primary endpoint was the change in HbA1c at week 24. The secondary endpoints included the change in body weight and discontinuation of oral semaglutide due to gastrointestinal adverse events at week 24.

### Statistical analysis

Patients co-administered with PPIs were matched to those without PPIs in a 1:1 ratio using propensity score matching with nearest-neighbor matching without replacement, with a caliper width of 0.2 of the standard deviation of the logit of the propensity score. Propensity scores were calculated using logistic regression analyses. The covariates included age, sex, baseline body weight, alanine aminotransferase, serum creatinine, and treatment pattern at the initiation of oral semaglutide, as well as baseline HbA1c and diabetes duration, which have been reported to influence the glycemic response to oral semaglutide in previous studies [12–14]. The balance of baseline characteristics between the matched groups was assessed using standardized mean differences (SMDs), with an SMD < 0.1 generally considered indicative of adequate balance. In the propensity score matched population, changes in HbA1c and body weight over time were analyzed using a linear mixed-effects model with a random intercept for individuals. The fixed effects included time, co-administration of PPIs, and their interactions. The oral semaglutide dose at week 24 and discontinuation of oral semaglutide due to gastrointestinal adverse events within 24 weeks were compared between the two groups using Fisher’s exact test. A two-sided *P* value was considered to be statistically significant. All statistical analyses were performed using EZR (Saitama Medical Center, Jichi Medical University, Saitama, Japan) version 2.7-1.

## Results

**Fig. 1:**
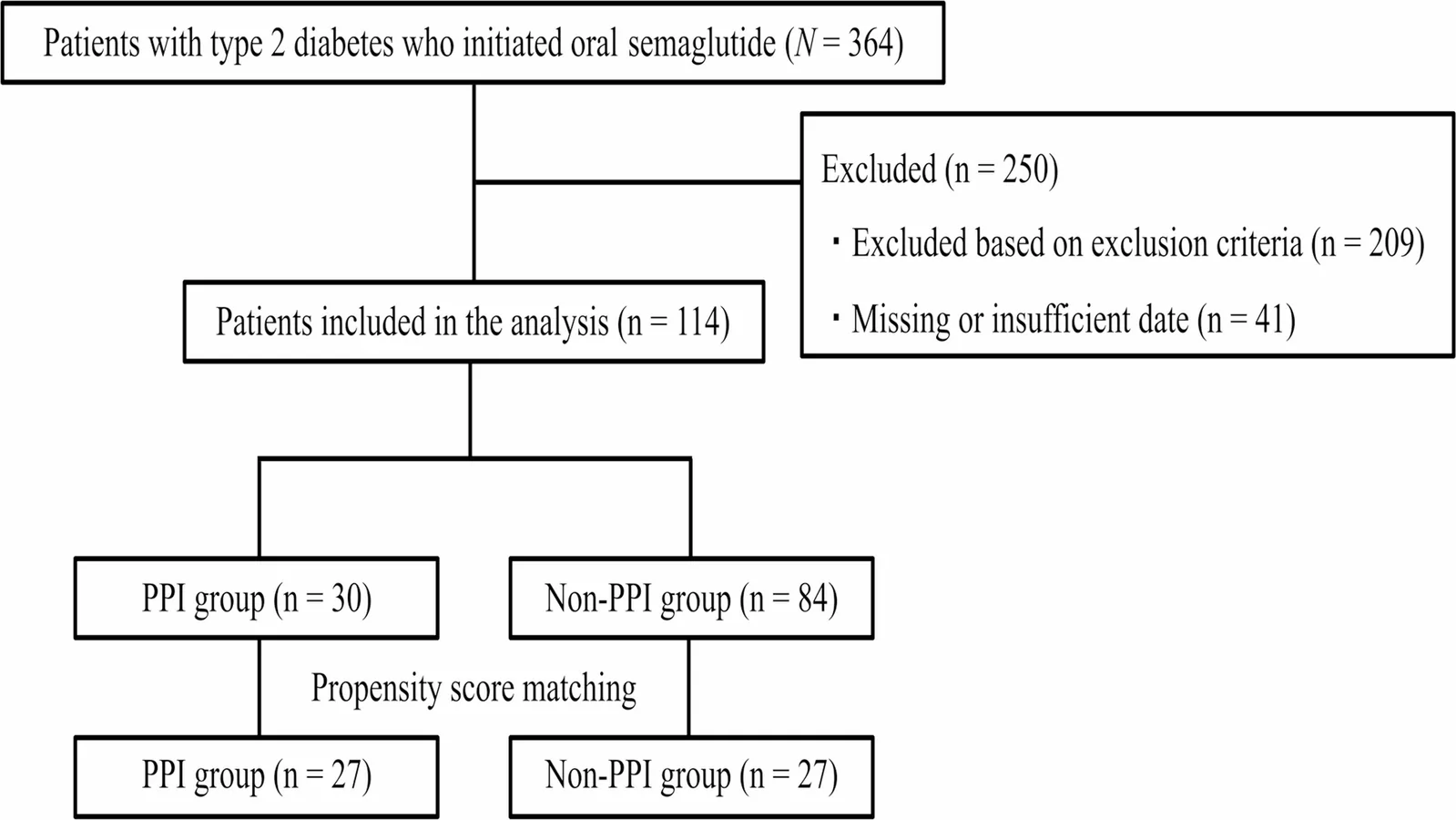
Flowchart of patient enrollment

Of the 364 patients with type 2 diabetes who initiated oral semaglutide, 114 met the inclusion criteria and were included in the analysis. Among them, 30 patients were co-administered PPIs. After propensity score matching, 27 matched pairs (54 patients) were included in the final analysis (Fig. 1).

Tables 1 and 2 show the baseline characteristics of the patients before and after propensity score matching, respectively. After matching, the baseline characteristics were generally well balanced between the two groups, although some residual imbalances remained (maximum SMD = 0.310), particularly for body mass index and selected concomitant medications.

**Table 1:** Demographics of patients at baseline before propensity score matching

Characteristic	All	PPIs group	Non-PPI group	SMD
Number of patients	114	30	84	
Age [years]	58.6 ± 12.6	64.7 ± 9.8	56.4 ± 12.8	0.728
Sex, female, n (%)	62 (54.4)	15 (50.0)	37 (44.0)	0.119
Body weight [kg]	72.8 ± 16.2	66.5 ± 11.6	75.0 ± 17.0	0.588
Body mass index [kg/ m^2^]	27.6 ± 5.1	26.3 ± 4.8	28.1 ± 5.1	0.353
Duration of diabetes [years]	11.0 ± 7.8	11.4 ± 7.3	10.9 ± 8.0	0.059
HbA1c [%]	8.1 ± 0.6	8.1 ± 0.6	8.1 ± 0.6	0.138
Serum creatinine [mg/dL]	0.69 ± 0.16	0.70 ± 0.15	0.68 ± 0.17	0.138
eGFR [mL/min/1.73m^2^]	84.7 ± 21.4	76.5 ± 13.6	87.7 ± 22.9	0.595
AST[IU/L]	27.6 ± 16.1	27.7 ± 11.7	27.6 ± 17.4	0.007
ALT [IU/L]	34.2 ± 29.2	29.9 ± 15.0	35.7 ± 32.7	0.227
Treatment pattern at initiation of oral semaglutide, n (%)				
Switched from other GLP1RA	21 (18.4)	4 (13.3)	17 (20.2)	0.186
Switched from DPP4 inhibitors	80 (70.2)	22 (73.3)	58 (69.0)	0.095
Switched from SGLT2 inhibitors	1 (0.9)	1 (3.3)	0 (0.0)	0.263
Add-on therapy	12 (10.5)	3 (10.0)	9 (10.7)	0.023
Concomitant medicine, n (%)				
Metformin	83 (72.8)	20 (66.7)	63 (75.0)	0.184
SGLT2 inhibitor	78 (68.4)	20 (66.7)	58 (69.0)	0.051
Sulphonyl-urea	23 (20.2)	7 (23.3)	16 (19.0)	0.105
Glinide	13 (11.4)	6 (20.0)	7 (8.3)	0.339
Insulin injection	20 (17.5)	7 (23.3)	13 (15.5)	0.200

Dates are expressed as the mean ± standard deviation or the number with percentage in parentheses

SMD, standardized mean difference; PPI, Proton pump inhibitor; HbA1c, Hemoglobin A1c; eGFR, estimated glomerular filtration rate; AST, Aspartate aminotransferase; ALT, Alanine aminotransferase; GLP1RA, Glucagon-like peptide-1 receptor agonist; DPP4, Dipeptidyl peptidase-4; SGLT2, Sodium-glucose cotransporter 2

**Table 2:** Demographics of patients at baseline after propensity score matching

Characteristic	All	PPIs group	Non-PPI group	SMD
Number of patients	54	27	27	
Age [years]	64.9 ± 8.5	64.8 ± 9.5	64.9 ± 7.6	0.009
Sex, female, n (%)	28 (51.9)	14 (51.9)	14 (51.9)	< 0.001
Body weight [kg]	66.0 ± 11.1	66.5 ± 12.0	65.9 ± 10.4	0.048
Body mass index [kg/ m^2^]	25.9 ± 4.1	26.5 ± 5.0	25.4 ± 3.0	0.277
Duration of diabetes [years]	12.2 ± 7.2	12.0 ± 6.9	12.3 ± 7.6	0.043
HbA1c [%]	8.1 ± 0.6	8.1 ± 0.6	8.1 ± 0.6	0.044
Serum creatinine [mg/dL]	0.69 ± 0.15	0.69 ± 0.15	0.69 ± 0.15	0.007
eGFR [mL/min/1.73m^2^]	77.8 ± 15.5	77.3 ± 13.9	78.4 ± 17.2	0.069
AST[IU/L]	26.6 ± 12.5	27.8 ± 12.1	25.5 ± 13.0	0.183
ALT [IU/L]	29.9 ± 15.2	30.3 ± 15.5	29.4 ± 15.1	0.056
Treatment pattern at initiation of oral semaglutide, n (%)				
Switched from other GLP1RA	6 (11.1)	3 (11.1)	3 (11.1)	< 0.001
Switched from DPP4 inhibitors	43 (79.6)	22 (81.5)	21 (77.8)	0.092
Switched from SGLT2 inhibitors	0 (0.0)	0 (0.0)	0 (0.0)	< 0.001
Add-on therapy	5 (9.3)	2 (7.4)	3 (11.1)	0.128
Concomitant medicine, n (%)				
Metformin	39 (70.4)	19 (70.4)	20 (74.1)	0.083
SGLT2 inhibitor	34 (63.0)	19 (70.4)	15 (55.6)	0.310
Sulphonyl-urea	11 (20.4)	6 (22.2)	5 (18.5)	0.092
Glinide	9 (16.7)	6 (22.2)	3 (11.1)	0.302
Insulin injection	10 (18.5)	5 (18.5)	5 (18.5)	< 0.01

Dates are expressed as the mean ± standard deviation or the number with percentage in parentheses

SMD, standardized mean difference; PPI, Proton pump inhibitor; HbA1c, Hemoglobin A1c; eGFR, estimated glomerular filtration rate; AST, Aspartate aminotransferase; ALT, Alanine aminotransferase; GLP1RA, Glucagon-like peptide-1 receptor agonist; DPP4, Dipeptidyl peptidase-4; SGLT2, Sodium-glucose cotransporter 2

The PPI used were lansoprazole (*n* = 13), vonoprazan (*n* = 9), and esomeprazole (*n* = 5). The distribution of oral semaglutide doses at week 24 did not differ significantly between the two groups (Table 3, *P* = 0.363).

**Table 3:** Distribution of oral semaglutide dose at 24 weeks

	All	PPIs group	Non-PPI group	*P*
Number of patients	40	19	21	
3 mg	10 (25.0)	6 (31.6)	4 (19.0)	0.363
7 mg	22 (55.0)	11 (57.9)	11 (52.4)	
14 mg	8 (20.0)	2 (10.5)	6 (28.6)	

Dates are expressed as the number with percentage in parentheses

*P* value is based on Fisher’s exact test

**Fig. 2:**
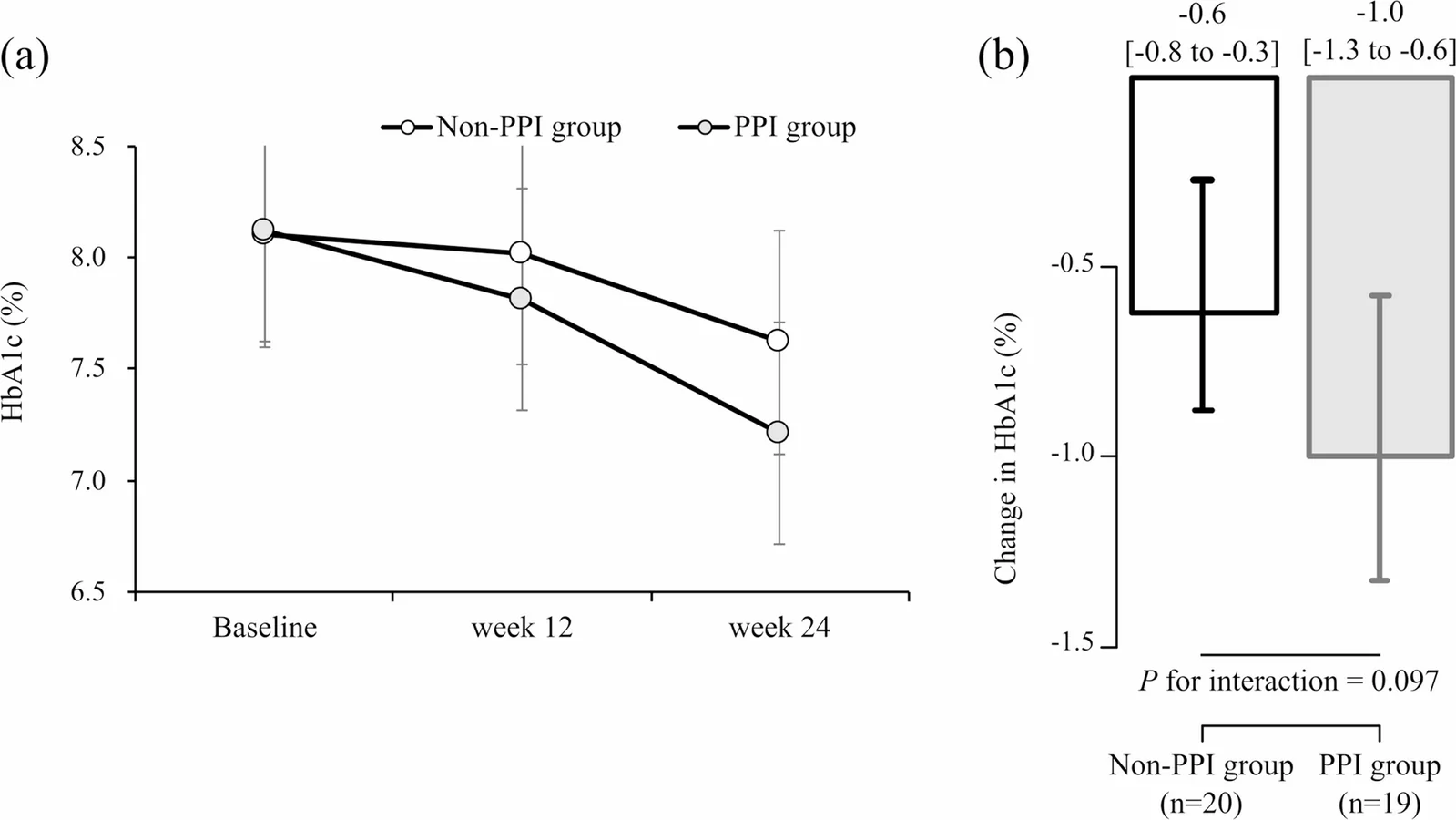
Data are presented as the mean [95% confidence interval]. (**a**) Changes in HbA1c at baseline, weeks 12, and 24. Gray circles represent the PPI group and white circles represent the non-PPI group. (**b**) Estimated mean change in HbA1c stratified by co-administration of PPIs (*P* for interaction = 0.097)

**Fig. 3:**
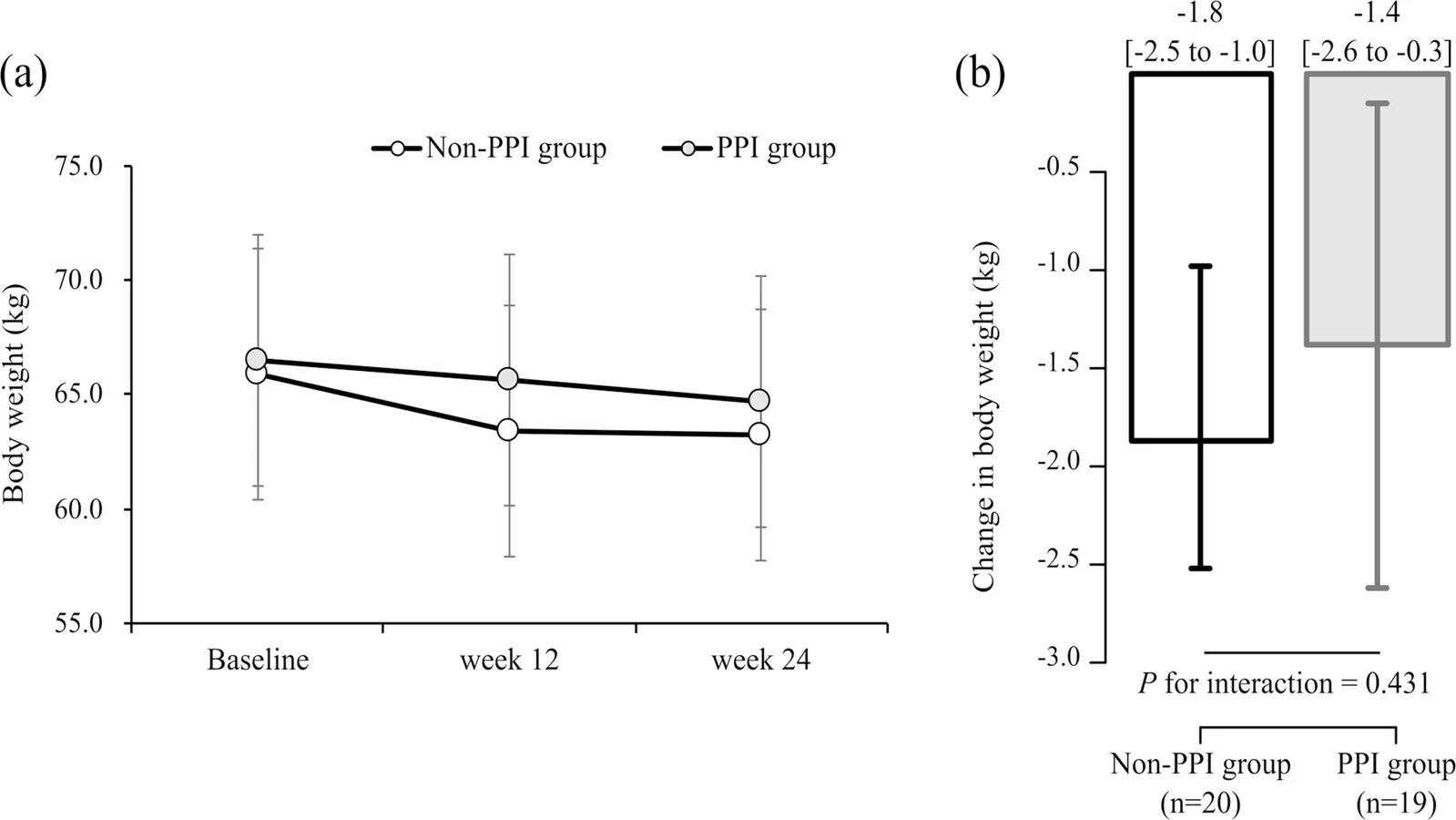
Data are presented as the mean [95% confidence interval]. (**a**) Changes in body weight at baseline, weeks 12, and 24. Gray circles represent the PPI group and white circles represent the non-PPI group. (**b**) Estimated mean change in body weight stratified by co-administration of PPIs (*P* for interaction = 0.431)

Changes in HbA1c and body weight from baseline to week 24 are shown in Figs. 2 and 3, respectively. The estimated mean (95% confidence interval (CI)) changes in HbA1c at week 24 was − 1.0% (-1.3 to -0.6) in the PPI group and − 0.6% (-0.8 to -0.3) in the non-PPI group, with no significant group-by-time interaction for HbA1c (*P* for interaction = 0.097). Similarly, the estimated mean (95% CI) changes in body weight at week 24 was − 1.4 kg (-2.6 to -0.3) in the PPI group and − 1.8 kg (-2.5 to -1.0) in the non-PPI group, with no significant group-by-time interaction (*P* for interaction = 0.431).

**Fig. 4:**
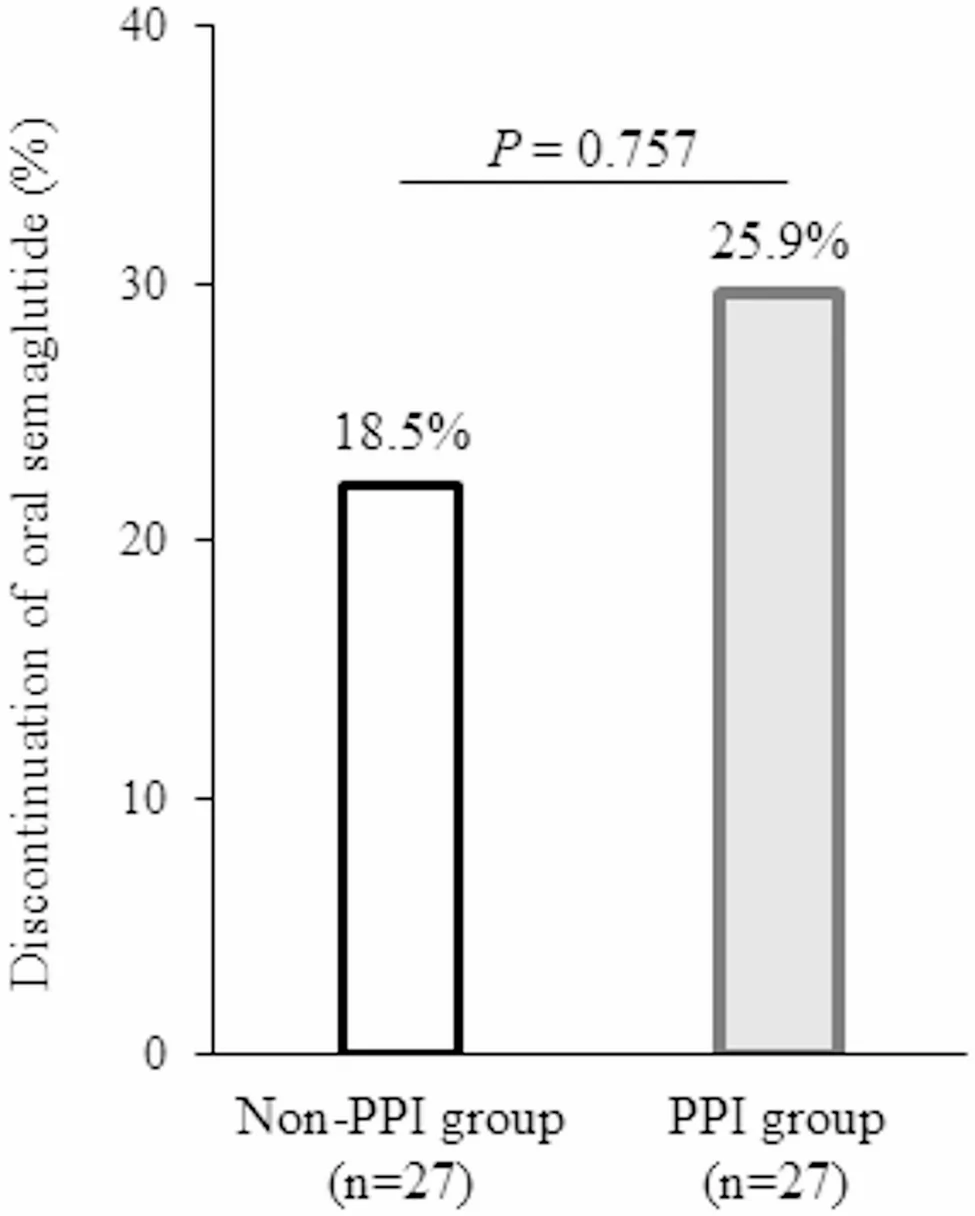
Discontinuation rates of oral semaglutide due to gastrointestinal adverse events within 24 weeks in the PPI and non-PPI groups

Discontinuation of oral semaglutide due to gastrointestinal adverse events within 24 weeks did not differ significantly between the groups (PPI vs. non-PPI: 25.9% vs. 18.5%, *P* = 0.757; Fig. 4). In addition, one patient in each group discontinued oral semaglutide at their own request, without gastrointestinal adverse events.

## Discussion

We investigated the effect of co-administration of PPIs on changes in HbA1c and body weight over 24 weeks in patients treated with oral semaglutide. The reduction in HbA1c observed in the non-PPI group was generally comparable to that reported in the PIONEER REAL Japan study [15, 16], supporting the validity of the glycemic response in the present study’s population. Although the reduction in body weight was somewhat smaller than that previously reported, both studies demonstrated clinically meaningful weight loss during oral semaglutide treatment. Differences in patient characteristics and routine clinical practices may have contributed to the observed differences in the magnitude of weight reduction. A recent exploratory study reported a significantly greater reduction in HbA1c at week 52 among patients receiving concomitant PPIs than among those without PPIs, whereas no significant between-group difference was observed at week 26 [10]. Because the first 26 weeks correspond to the primary efficacy assessment used in the pivotal PIONEER clinical trial program and represent a clinically meaningful period for evaluating the early efficacy of oral semaglutide [3], this present study aimed to provide a more rigorous evaluation of the findings at week 24 by applying a longitudinal mixed-effects model and propensity score matching. This approach enabled us to reassess the short-term clinical impact of the co-administration of PPI while minimizing the influence of baseline confounding.

Overgaard et al. reported that the glucose-lowering effect of oral semaglutide depends on systemic semaglutide exposure [12]. Oral semaglutide contains SNAC, which enhances semaglutide absorption through multiple complementary mechanisms, including local buffering of gastric acidity, protection from enzymatic degradation, and enhancement of transcellular absorption by increasing membrane fluidity [3, 4]. Baekdal et al. reported that the co-administration of a PPI for 10 days increased semaglutide exposure by approximately 15% in healthy volunteers, although it was not statistically significant [9]. However, this pharmacokinetic study was conducted in healthy Western volunteers in controlled conditions. Therefore, these findings should be interpreted with caution when extrapolating them to Japanese patients with type 2 diabetes who receive oral semaglutide in routine clinical practice. Differences in study populations, disease status, and clinical treatment conditions may influence the clinical relevance of pharmacokinetic interactions. Although the pharmacokinetic interaction observed by Baekdal et al. appeared modest, additional mechanisms beyond the modest increase in semaglutide exposure have been proposed. Mastunuma et al. proposed several mechanisms that may enhance the glucose-lowering effect of oral semaglutide during concomitant PPI therapy, including suppression of pepsin activity, reduced gastric fluid volume, and delayed gastric emptying [10]. However, these proposed mechanisms may not necessarily translate into clinically measurable differences in glycemic control during the first 24 weeks of treatment with semaglutide. This may partly explain why no significant between-group differences in glycemic control were observed during the first 24 weeks in the present study.

Our findings are consistent with the absence of a significant between-group difference during the earlier phase reported in a previous study [10]. Baseline HbA1c and diabetes duration, which have been reported to influence the glucose-lowering effect of oral semaglutide [13, 14], were generally comparable between the two studies. Differences in these variables alone are unlikely to account for the similar findings observed during the first 24–26 weeks. Taken together, these findings suggest that the impact of potential drug-drug interactions involving PPIs on the efficacy of oral semaglutide may be limited during the first 24 weeks of treatment.

Consistently, no significant association was observed between changes in body weight and co-administration of PPIs in the present study. Although the weight-reducing effect of oral semaglutide has been reported to be dependent on its plasma concentration [12], the mechanisms underlying weight reduction differ from those responsible for glucose lowering effects. GLP-1 RAs reduce glucose levels through insulinotropic effects and glucagon secretion suppression [17]. In contrast, body weight reduction is primarily mediated by delayed gastric emptying and central effects on appetite regulation in the hypothalamus [17], which may introduce greater variability and make the effect less directly associated with drug exposure. In addition, recent studies have suggested that eating behavior patterns are associated with weight reduction in patients treated with GLP-1 RAs [18]. As dietary intake was not assessed in the present study, this factor may have contributed to variability in body weight changes. Taken together, these findings may partly explain the lack of association between PPI co-administration and the body weight changes observed in the present study.

No significant association was observed between the co-administration of PPIs and discontinuation of semaglutide due to gastrointestinal adverse events within 24 weeks in the present study. Gastrointestinal symptoms, such as nausea and vomiting, associated with GLP-1 RAs are thought to result from delayed gastric emptying and central effects mediated by GLP-1 receptors [17]. The onset of these symptoms is dependent on plasma semaglutide exposure [12]. In addition, Shiomi et al. reported that co-administration of PPIs was a risk factor for gastrointestinal adverse events with other GLP-1 RA (liraglutide/lixisenatide), although the underlying mechanism remains unclear [19]. However, these adverse events are likely mediated by multiple physiological pathways, including both peripheral and central mechanisms, and are not solely determined by systemic drug exposure but may also be affected by patient- and treatment-related factors. Moreover, as discontinuation was used as the endpoint in this study, the findings may primarily reflect clinically significant or persistent symptoms rather than the overall incidence of mild or transient adverse events in the general population.

This study had several limitations that should be considered. Although propensity score matching improved the balance of baseline characteristics, some residual imbalances remained, particularly in body mass index and selected concomitant medications, including glinides and sodium-glucose cotransporter 2 inhibitors, which may have influenced the observed treatment effects. Additional adjustments for these residual imbalances were not performed; therefore, their potential impacts cannot be completely excluded. This was a single-center retrospective study with a relatively small sample size, which may limit the generalizability of the findings and increase the possibility of type Ⅱ error. In addition, subgroup analyses according to individual PPIs could not be performed because the number of patients receiving each PPI was limited. Furthermore, as this was a retrospective study, important factors affecting semaglutide exposure, such as post-dose fasting duration and volume of co-administered water, were not assessed [20]. These limitations should be considered when interpreting the findings of the present study. Further prospective studies incorporating pharmacokinetic assessments are warranted to elucidate the relationship between PPI co-administration and the efficacy and safety of oral semaglutide.

## Conclusions

24-week co-administration of PPIs was not associated with glycemic control, body weight changes, or discontinuation due to gastrointestinal adverse events in patients receiving oral semaglutide.

## Data Availability

No datasets were generated or analysed during the current study.

## Abbreviations

PPI: Proton pump inhibitor
GLP-1RA: Glucagon-like peptide-1 receptor agonist
SNAC: Sodium N-[8-(2-hydroxybenzoyl) amino] caprylate
BMI: Body mass index
HbA1c: Hemoglobin A1c
eGFR: estimated glomerular filtration rate
AST: Aspartate aminotransferase
ALT: Alanine aminotransferase
DPP4: Dipeptidyl peptidase-4
SGLT2: Sodium-glucose cotransporter 2
